# pERK1/2 silencing sensitizes pancreatic cancer BXPC-3 cell to gemcitabine-induced apoptosis via regulating Bax and Bcl-2 expression

**DOI:** 10.1186/s12957-015-0451-7

**Published:** 2015-02-21

**Authors:** Min Wang, Xingjiao Lu, Xueguang Dong, Fengyun Hao, Zimin Liu, Guangzhen Ni, Dong Chen

**Affiliations:** Department of Clinical Laboratory, People’s Hospital of Laiwu, Laiwu, Shangdong China; Department of Internal Neurology, People’s Hospital of Zhangqiu, ZhangQiu, Shangdong China; Department of General Surgery, The Affiliated Hospital of Qingdao University, Qingdao, China; Department of Clinical Laboratory, People’s Hospital of Weifang, Weifang, Shangdong China

**Keywords:** Pancreatic cancer, ERK1/2, Bcl-2, Bax, Gemcitabine

## Abstract

**Background:**

Our previous study has demonstrated that knockdown of activated ERK1/2(pERK1/2) sensitizes pancreatic cancer cells to chemotherapeutic drug gemcitabine (Gem) treatment. However, the details of this survival mechanism remain undefined. It has also shown that Bcl-2 confers resistance and Bax sensitizes to gemcitabine-induced apoptosis in pancreatic cancer cells. Furthermore, the extracellular signaling-regulated kinase (ERK) signaling pathway regulates Bcl-2/Bax expression ratio. We therefore tested the hypothesis that pancreatic cancer cells are resistant to gemcitabine and this resistance is due to activation of ERK1/2 and subsequent upregulation of Bcl-2 and downregulation of Bax.

**Methods:**

Pancreatic cancer cell BXPC-3 was used in the study. The effect of pharmacological inhibition of ERK1/2 on resistance of pancreatic cancer cells to apoptosis induced by treatment with gemcitabine was analyzed. The following methods were utilized: TUNEL and ELISA were used to detect apoptosis. Western blot was used to detect the protein expression.

**Results:**

Gemcitabine treatment enhanced the activity of ERK1/2 in the BXPC-3 cells. Inhibition of the ERK1/2 by PD98059 could downregulate Bcl-2 and upregulate Bax and was associated with restoration of sensitivity to gemcitabine in BXPC-3 cells. Depletion of endogenous Bcl-2 expression by specific small interfering RNA transfection significantly increased gemcitabine-induced cell apoptosis. Combined treatment with PD98059 and Bax siRNA transfection could decrease gemcitabine-induced ERK1/2 and Bax activation, which subsequently resulted in decreased apoptosis.

**Conclusions:**

The upregulation of ERK1/2-dependent Bcl-2 and downregulation of ERK1/2-dependent Bax can protect human pancreatic cancer cells from gemcitabine-induced apoptosis. Targeting the ERK1/2-Bax/Bcl-2 pathway may in part lead to sensitization of pancreatic cancer to gemcitabine.

## Background

Pancreatic cancer is a highly lethal malignancy resistant to the apoptosis-inducing effects of radio- and chemotherapy [1]. Although gemcitabine monotherapy, a deoxycytidine analogue, or its combination with other agents has become standard chemotherapy for the treatment of advanced pancreatic cancer [2-5], it failed to significantly improve the outcome of pancreatic carcinoma patients, because of preexisting or acquired chemoresistance of most of the tumor cells [6]. Chadha et al. [7] have reported that activated ERK1/2(pERK1/2) was overexpressed in pancreatic carcinoma. Our recent study has demonstrated that knockdown of pERK1/2 sensitizes pancreatic cancer cell lines to gemcitabine treatment [8]. However, the details of this survival mechanism remain undefined.

Apoptosis is a cell death process that plays a critical role in mammalian development and tissue homeostasis. It has now become clear that apoptosis is also the mechanism of tumor cell death in response to a variety of chemotherapeutic agents. The Bcl-2 family of proteins plays a key role in the regulation of apoptosis. Some members of this family, including Bax, Bak, Bid, and Bik, function as proapoptotic factors, and others, including Bcl-2, Bcl-xL, Mcl-1, and A1, function as antiapoptotic proteins. Members of the Bcl-2 family share regions of homology called BH1 and BH2, and a hydrophobic C-terminus which serves to anchor the proteins to the membrane. Two additional conserved domains, BH3 and BH4, are found only among some members of the Bcl-2 family. These domains are involved in homo- and heterodimerization of Bcl-2-like proteins. While there are different ways in which Bcl-2 members may function, the ratio of heterodimerization between anti- and proapoptotic factors has been suggested to be a pivotal decision point for death or survival [9-12]. It has previously shown that Bcl-2 confers resistance to gemcitabine-induced apoptosis in pancreatic cancer cells, and siRNA-mediated silencing of anti-apoptotic Bcl-2 enhances chemotherapy sensitivity in human pancreatic cancer cells *in vitro* and might lead to improved therapy responses in advanced stages of this disease [13].

Bax, a proapoptotic factor, contains BH1 and BH2 domains, as well as the BH3 domain which is important for heterodimerization with Bcl-2 and Bcl-xL factors. Overexpression of the Bax gene has been found to induce apoptotic death in pancreatic cancer cells [14,15].

Several studies have found that constitutive activation of the extracellular signaling-regulated kinase (ERK) could induce Bcl-2 upregulation and Bax downregulation [16-18]. We therefore suggested that ERK might be involved in regulation of Bcl-2/Bax signals.

In the present study, we demonstrate that the ERK1/2-Bcl-2/Bax signaling pathway is a key regulator of gemcitabine chemoresistance in pancreatic cancer BXPC-3 cells. And knockdown of ERK1/2 could sensitize BXPC-3 cells to gemcitabine chemotherapy through modulating Bcl-2/Bax pathway. These results provide possible routes for therapeutic intervention for pancreatic cancer.

## Methods

### Agents

The following primary and secondary antibodies were purchased from Cell Signaling Technology Inc. (Shanghai, China): Anti-ERK1/2, Anti-β-actin, Anti-Bcl-2, and Anti-Bax. Dimethyl sulfoxide (DMSO) was bought from AppliChem GmbH (Ottoweg4, D-64291 Darmstadt, Germany). Fetal bovine serum (FBS) and penicillin-streptomycin were acquired from Invitrogen (Carlsbad, CA, USA). PD98059 were purchased from Calbiochem Corp. (San Diego, CA, USA).

### Cell culture

The human pancreatic adenocarcinoma cell lines BXPC-3 were obtained from the American Type Culture Collection (Rockville, MD, USA).The BXPC-3 cell line has been previously demonstrated to be resistant to gemcitabine-induced apoptosis [6]. Cells were routinely cultured in DMSO supplemented with 10% fetal bovine serum in a 37°C incubator in a humidified atmosphere of 5% CO_2_. The medium was refreshed every 2 days. Cells were trypsinized by trypsin-EDTA. The cells in the logarithmic growth phase were used to conduct the experiments described as follows. All experiments were done in triplicate.

### Cell treatment

To determine the effect of gemcitabine on apoptosis of BXPC-3 cells, the cells were seeded for 24 h, then treated with 0–25 μM for 72 h. To determine the effect of ERK1/2 inhibition on gemcitabine-induced apoptosis of BXPC-3, the cells were treated with 25 μM PD98059 for 24 h, then treated with 0–25 μM gemcitabine for 72 h.

To determine the effect of Bax on gemcitabine-induced apoptosis of BXPC-3, the cells were treated with 25 μM PD98059 and 15 μM anti-Bax antibody for 24 h, then treated with 0–25 μM gemcitabine for 72 h.

### siRNA transfection

The sense-strand sequences of Bcl-2 small interfering RNA (Bcl-2 siRNA) or Bax small interfering RNA (Bax siRNA) and control siRNA used were purchased from Shanghai, China. BXPC-3 cells were transfected with siRNA duplexes (200 nM) with Lipofectamine 2000 (Invitrogen, Carlsbad, CA) for 24 h according to the manufacture’s instruction.

### Quantification of apoptosis by ELISA

The Cell Apoptosis ELISA Detection Kit (Roche, Palo Alto, CA) was used to detect apoptosis in BXPC-3 cells with different treatments above according to the manufacturer’s protocol. After treatment, the cytoplasmic histone DNA fragments from BXPC-3 cells with different treatments were extracted and bound to immobilize anti-histone antibody. Subsequently, the peroxidase-conjugated anti-DNA antibody was used for the detection of immobilized histone DNA fragments. After addition of substrate for peroxidase, the spectrophotometric absorbance of the samples was determined using ULTRA Multifunctional Microplate Reader (TECAN) at 405 nm.

### Apoptosis assay by TUNEL

Terminal deoxynucleotidyl transferase-mediated deoxyribonucleotide triphosphate nick end-labeling (TUNEL) was generally used to assess cell death in BXPC-3 cells with different treatments above according to the manufacturer’s protocol. The cells were fixed with 4% paraformaldehyde for 30 min at room temperature. Thereafter, the cells were incubated with the TUNEL reaction mixture (Roche Molecular Biochemicals, Indianapolis, IN) for 60 min at 37°C followed by labeling with fluorescein isothiocyanate (FITC)-conjugated anti-fluorescein anti-goat antibody (Fab fragment) for an additional 30 min. The nuclei were counterstained with 4,6-diamidino-2-phenylindole (DAPI). Finally, TUNEL-positive cells were photographed on an Olympus microscope.

### Western blot assay

Total cellular proteins were isolated, and the protein concentration of the sample was determined by Bio-Rad DC Protein Assay (Bio-Rad Laboratories Inc., Hercules, CA). Bcl-2, Bax, and β-actin were detected. The targeted protein was revealed by enhanced chemiluminescence (ECL).The membrane was incubated with an ECL solution (Biological Industries) and exposed to ECL film (Eastman Kodak, Rochester, NY) to visualize specifically labeled proteins. The resulting exposed films were then analyzed by densitometry. All experiments were performed at least three times.

### Measurement of ERK activation

BXPC-3 cells were treated with 0–25 uM gemcitabine. For 0–72 h, the cell layers were washed twice with cold PBS and then lysed with a buffer consisting of 20 mM Tris–HCl (pH 7.5), 150 mM NaCl, 1% Triton X-100, 10 mM NaH_2_PO_4_, 10% glycerol, 2 mM Na_3_VO_4_, 10 mM NaF, 1 mM ABSF, 10 μg/mL leupeptin, and 10 μg/mL aprotinin. Western blot analysis was carried out as above. Equal amount of proteins was transferred onto PVDF membranes, then incubated with anti-ERK or anti-pERK monoclonal antibodies (1:500). The ECL detection kit was used for detection.

### Statistical assessment

All statistical analyses were performed using SPSS 13.0 software. The results were presented as mean ± SD of three replicate assays. Differences between various groups were assessed using ANOVA or *t*-test. A *P* value of <0.05 was considered to indicate statistical significance.

## Results

### Gemcitabine-activated ERK signaling pathway in BXPC-3 cells

**Figure 1:**
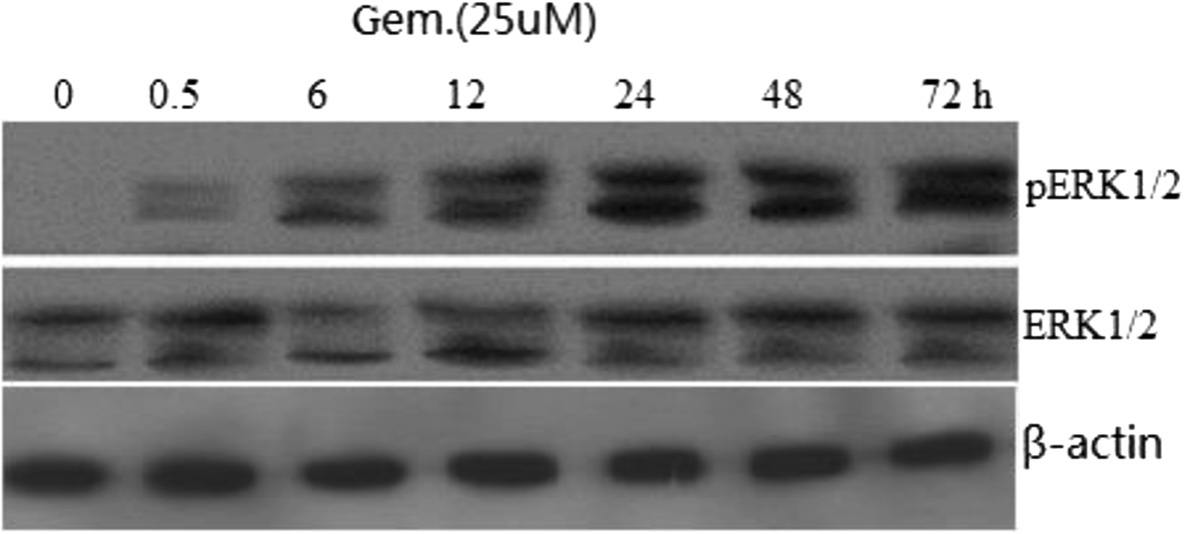
**Effects of gemcitabine on ERK signaling pathways in BXPC-3.** Anti-ERK and anti-pERK1/2 monoclonal antibodies were used to perform in Western blotting. Cells were exposed to 25 uM gemcitabine for 0–72 h. The levels of ERK1/2 and pERK1/2 were measured by densitometry of autoradiographs.

ERK signaling cascade is a classic pathway involved in the regulation of cell death. We confirmed that the BXPC-3 cell line demonstrates the less activation of ERK1/2 under basal conditions by Western blotting with anti-ERK1/2 and anti-PERK1/2 monoclonal antibodies (Figure 1). Western blotting showed that the levels of pERK1/2 were upregulated after 30-min incubation with 25 uM gemcitabine compared with the control group. Additionally, this effect was time-dependent with the peak pERK1/2 at 24 h of incubation. However, total ERK1/2 did not change after treatment with gemcitabine (Gem).

### ERK1/2 activation is correlated with enhanced time-dependent protein levels of Bcl-2 and decreased Bax levels induced by gemcitabine

**Figure 2:**
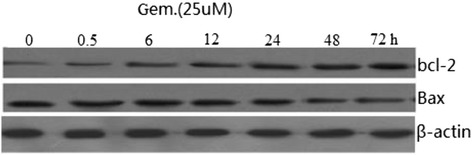
**Effects of Gem on the expression of Bcl-2 and Bax in BXPC-3.** Cells were treated with 25 uM gemcitabine for 72 h before collecting proteins. Cell lysates were subjected to Western blot analysis and incubated with anti-Bax, anti-Bcl-2, or anti-β-actin monoclonal antibodies. The gel image analysis software was used to analyze the absorbance values of Bcl-2 and Bax.

Bcl-2 and Bax are the essential members of Bcl-2 family which involved in the process of apoptosis. Western blot analysis was used to detect the expression of Bcl-2 and Bax in BXPC-3 cells incubated with 25 uM gemcitabine for 0–72 h. As a result, we found that gemcitabine increased the levels of Bcl-2 protein, while it decreased the expression of Bax in BXPC-3 cells. Bcl-2 expression changes as the same way as pERK1/2, but Bax expression changes the opposite way as pERK1/2 (Figure 2).

### ERK1/2 activation is correlated with enhanced dose-dependent protein levels of Bcl-2 and decreased Bax protein levels induced by gemcitabine

**Figure 3:**
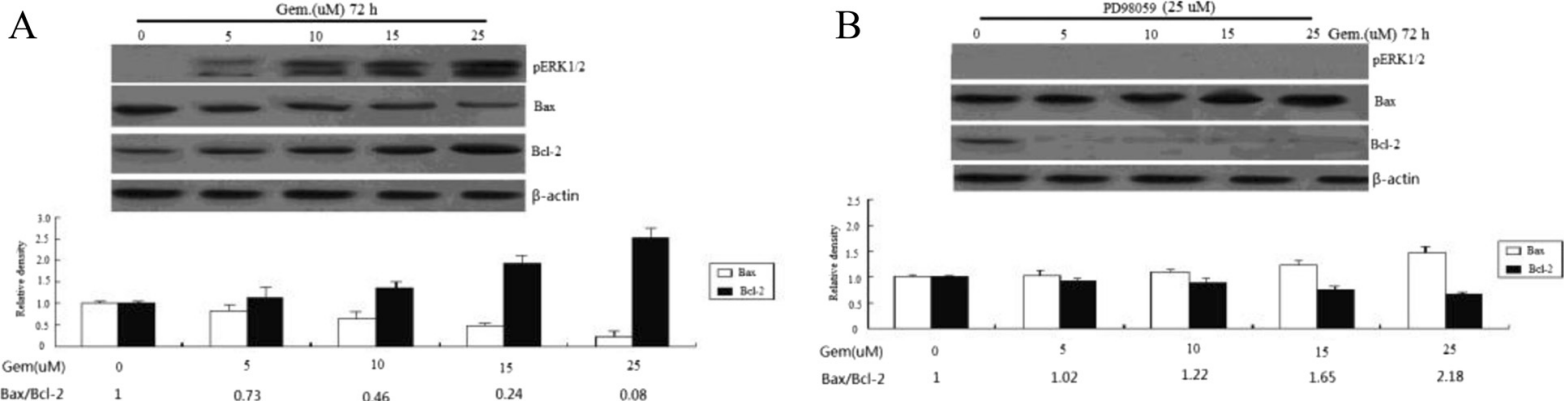
**Effects of Gem on the expression of Bcl-2 and Bax in BXPC-3. (A)** Cells were treated with 0–25 uM gemcitabine for 72 h before collecting proteins. **(B)** BXPC-3 cells were treated with 25 μM PD98059 for 24 h, then treated with 0–25 μM gemcitabine for 72 h. Cell lysates were subjected to Western blot analysis and incubated with anti-pERK1/2, anti-Bax, anti-Bcl-2, or anti-β-actin monoclonal antibodies. The gel image analysis software was used to analyze the absorbance values of Bcl-2 and Bax.

Bcl-2 and Bax are the essential members of Bcl-2 family which involved in the process of apoptosis. Western blot analysis was used to detect the expression of Bcl-2 and Bax in BXPC-3 cells incubated with 0–25 uM gemcitabine for 72 h. As a result, we found that gemcitabine increased the levels of pERK1/2 in a dose-dependent, and it also increased the levels of Bcl-2 protein, while it decreased the expression of Bax in BXPC-3 cells in a dose-dependent manner (Figure 3A, all *P* < 0.05). The Bax/Bcl-2 ratio was set to one in the control group, and 25 uM gemcitabine could downregulate the Bax/Bcl-2 ratio with a maximal decrease to 0.08 (Figure 3A, all *P* < 0.05).

To determine whether the changes of Bcl-2 and Bax were mediated by alterations in pERK1/2, BXPC-3 cells were treated with 25 μM PD98059 for 24 h, then treated with 0–25 μM gemcitabine for 72 h. The results showed that, following treatment with PD98059, a dose-dependent reduction in Bcl-2 expression was observed in the BXPC-3/gemcitabine cells and a dose-dependent increase in Bax expression was observed in the BXPC-3/gemcitabine cells (Figure 3B). A parallel reduction in pERK1/2 activation was observed following ERK1/2 inhibition with PD98059 in the BXPC-3 cells treated with gemcitabine (Figure 3B). The Bax/Bcl-2 ratio was set to one in the control group, and PD98059 could upregulate the Bax/Bcl-2 ratio with a maximal increase to 2.18 (Figure 3B).

### ERK1/2 inhibition promote Gem-induced cell apoptosis

**Figure 4:**
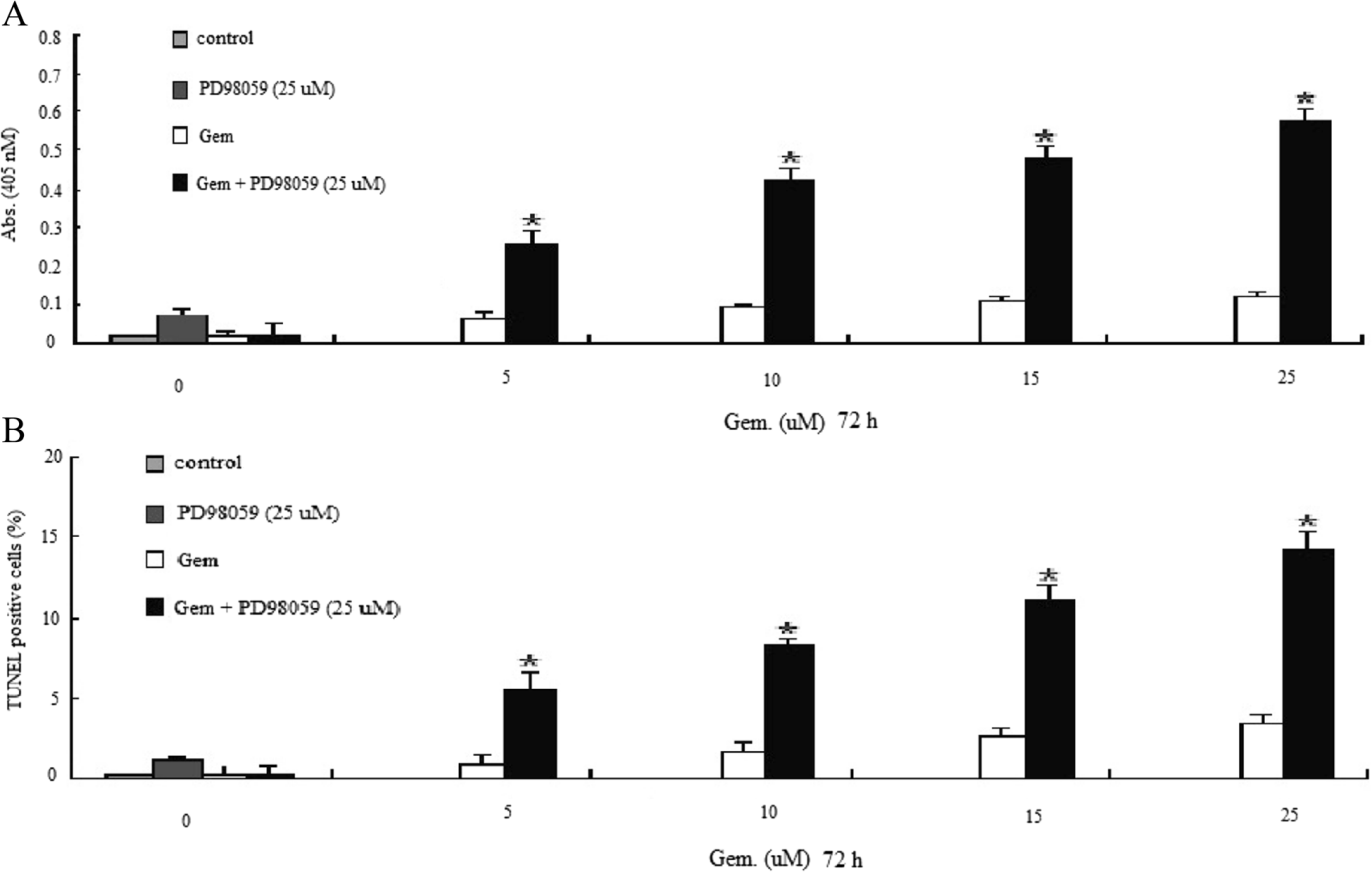
**Evaluation of cell apoptosis by PD98059 pretreatment using ELISA technique in pancreatic cancer BXPC-3 cells.** Sensitization of pancreatic tumor BXPC-3 cells to PD98059- and/or gemcitabine-induced apoptotic cell death by ELISA assay **(A)** and TUNEL assay **(B)** after 24 h of pretreatment with 25 μM PD98059, 0–25 μM gemcitabine, or combination of PD98059 and 0–25 μM gemcitabine for 72 h. Increased apoptotic response was evident in the combination treatment group of the cells relative to untreated control or individual treatment groups; *P < 0.05, statistical significance.

We observed induction of apoptosis in pancreatic cancer BXPC-3 cells treated with either 25 μM PD98059 or 0–25 μM gemcitabine alone. Relative to single agents, PD98059 pretreatment followed by gemcitabine treatment induced much more apoptosis in the BXPC-3 cell lines as shown by both histone DNA ELISA (Figure 4A) as well as TUNEL analysis (Figure 4B). These results are consistent with our previous study [8], suggesting that ERK1/2 activity may protect pancreatic cancer cells from gemcitabine-induced apoptosis, and ERK1/2 silencing sensitized BXPC-3 cells to Gem-induced apoptosis.

### Knockdown of Bcl-2 expression by siRNA transfection sensitizes BXPC-3 cells to gemcitabine-induced apoptosis

**Figure 5:**
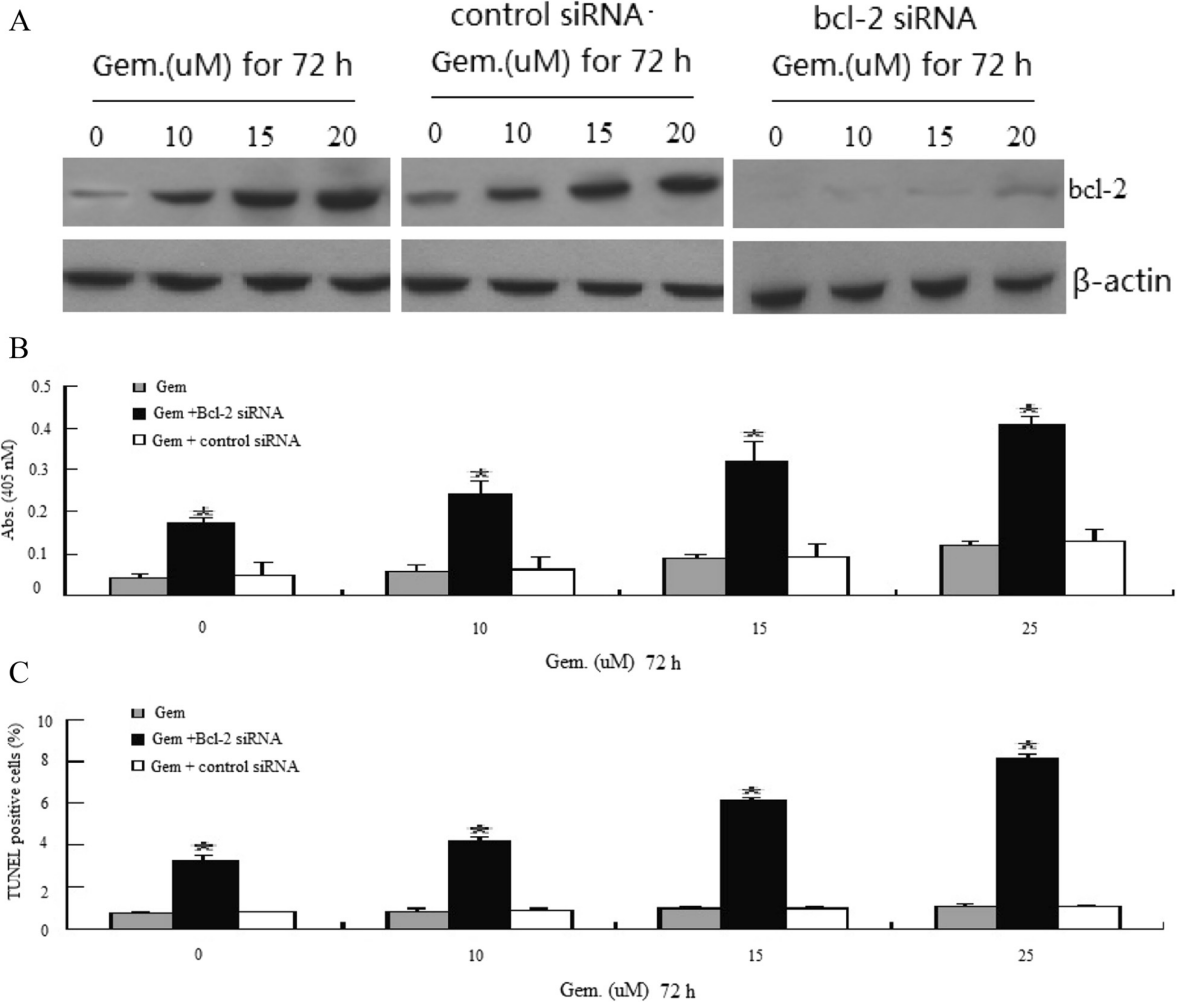
**Modulation of sensitivity to Gem by ERK1/2-Bcl-2 signal.** BXPC-3 cells were transfected with siRNA duplexes (200 nM) specific to Bcl-2 or scrambled control in complete medium for 24 h before treatment with gemcitabine (0–25 uM) for 72 h. **(A)** Western blot assay for Bcl-2 expression in BXPC-3 cells; **(B)** cell apoptosis was determined using the ELISA assay; and **(C)** cell apoptosis was determined using the TUNEL assay. The results (mean ± SEM) were from four independent experiments. *P < 0.05.

To directly address the role of Bcl-2 in gemcitabine resistance, the effects of Bcl-2 knockout by siRNA transfection on gemcitabine-induced apoptosis were determined by TUNEL and ELISA assays. Total levels of Bcl-2 in cells transfected with specific siRNA decreased significantly compared with mock-transfected cells, indicating that Bcl-2 siRNA was sufficient to knockdown Bcl-2 expression (Figure 5A). Furthermore, gemcitabine-induced ERK1/2 phosphorylation was not affected in cells transfected with Bcl-2 siRNA (data not shown), suggesting that ERK1/2 indeed was the upstream signal in regulating Bcl-2 expression induced by gemcitabine. The apoptosis was assessed using the TUNEL and ELISA assays (Figure 5B,C). The suppression of Bcl-2 protein expression by small interfering Bcl-2 RNA markedly increased the sensitivity of cells to gemcitabine compared with small interfering control RNA-transfected cells (Figure 5B,C). Taken together, these data suggest that in human BXPC-3 cells, Bcl-2 is a pivotal protein for affecting drug resistance to gemcitabine.

### Inhibition of Bax by Bax siRNA suppressed PD98059 and gemcitabine cotreatment-induced apoptosis

It has showed above that gemcitabine and PD98059 cotreatment promoted apoptosis followed by increased Bax activation. We next investigated whether Bax inactivation would inhibit the apoptosis effect induced by gemcitabine and PD98059 cotreatment.

**Figure 6:**
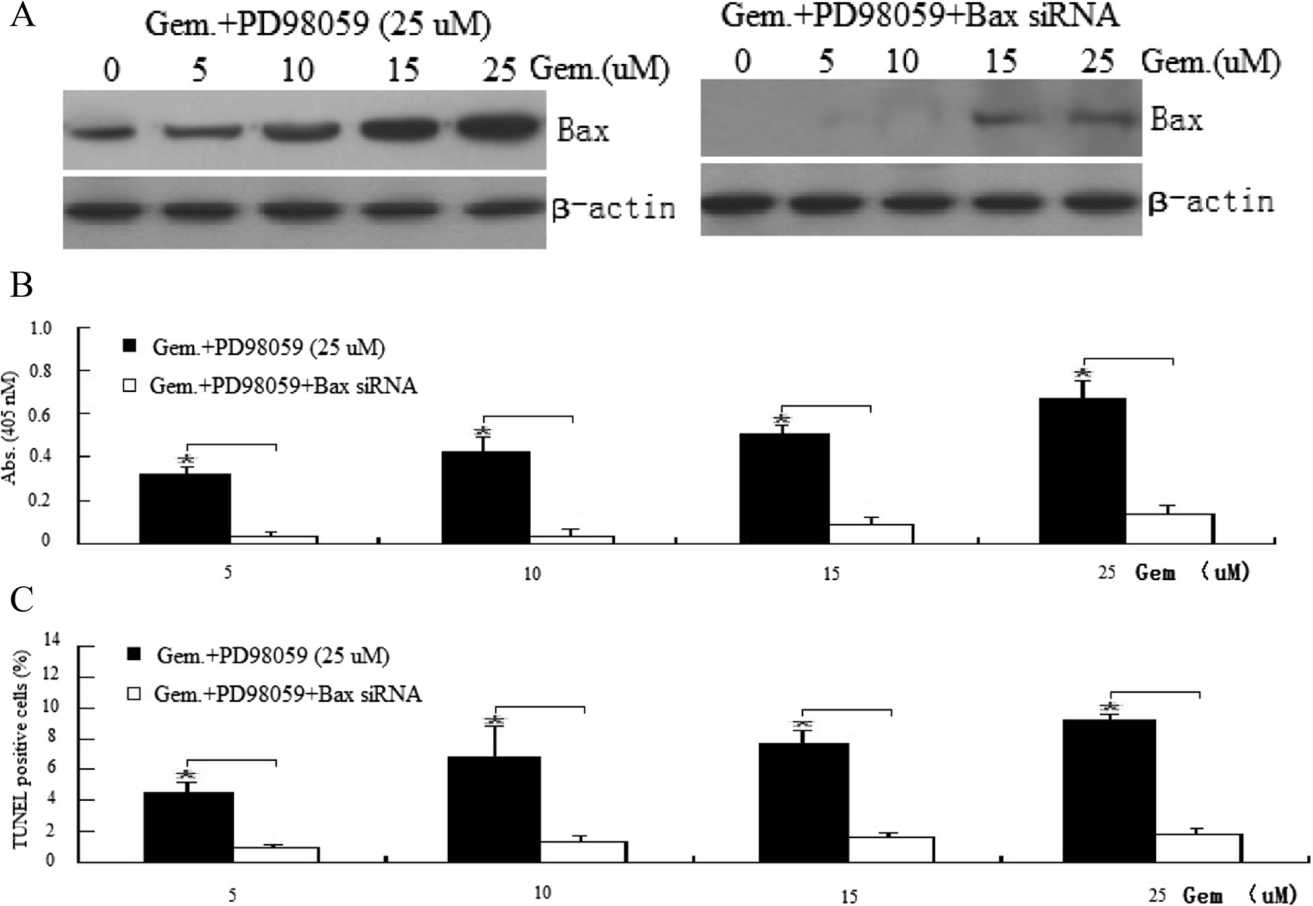
**Modulation of sensitivity to gemcitabine by ERK1/2-Bax signal.** Cells were pre-cotreated with PD98059 and Bax siRNA for 24 h and then treated with 0–25 uM gemcitabine for 72 h. **(A)** Western blot assay for Bax expression in BXPC-3 cells; **(B)** cell apoptosis was determined using the ELISA; and **(C)** cell apoptosis was determined using the TUNEL assay. *P < 0.05.

Total levels of Bax in cells transfected with Bax siRNA decreased significantly compared with control siRNA cells, indicating that Bax was significantly inhibited (Figure 6A). Furthermore, gemcitabine-induced ERK1/2 phosphorylation was not affected in cells transfected with Bax siRNA (data not shown). When cells were cotreated with PD98059 and Bax siRNA for 24 h, the TUNEL and ELISA assays showed a marked decrease in cell apoptosis treated with gemcitabine (Figure 6B, C). Control siRNA did not have significant effect on gemcitabine-PD98059-induced apoptosis (data not shown).These results indicate that ERK1/2 has protective effects against Gem-induced apoptosis by downregulation of Bax.

## Discussion

Inhibition of the ERK1/2 has been shown to sensitize pancreatic cancer cells *in vitro* and *in vivo* to the apoptotic effect of chemotherapy [8]. The mechanism by which ERK1/2 activation in these cancer cells confers that chemoresistance is unclear.

The cell intrinsic apoptosis pathway is regulated by the Bcl-2 proteins, which control the release of cytochrome c from mitochondria, triggering the activation of caspases and cell death. The Bcl-2 family consists of pro-survival and prodeath proteins that share a number of Bcl-2 homology (BH) domains (BH1–4) [19]. In the pro-survival proteins (such as Bcl-2, Bcl-xL, and Mcl-1), the BH1–3 domains form a hydrophobic pocket that can bind the BH3 domains of certain other family members. The multi-domain pro-apoptotic proteins (Bax and Bak) also use BH1–3 to form a hydrophobic pocket, and many viable cells are found in complex with pro-survival Bcl-2 proteins. Bax and/or Bak are normally sequestered by pro-survival Bcl-2 proteins in viable cells. In response to stress-induced signals, Bak/Bax was released from Bcl-2, which can then oligomerize, causing mitochondrial outer membrane permeabilization and release of cytochrome c.

There is abundant evidence that survival factors can use the ERK1/2 pathway to increase the expression of several pro-survival Bcl-2 proteins, notably Bcl-2, Bcl-xL, and Mcl-1, by promoting *de novo* gene expression in a variety of cell types, for example, MEK inhibition caused a decrease in Bcl-2, Bcl-xL, and Mcl-1 and apoptosis in pancreatic cancer cells [20]. Pro-apoptosis protein was also regulated by Bax [16-18].

In this study, we reported that Bcl-2 and Bax are important determinants of drug resistance to gemcitabine in BXPC-3 cells. Gem induces Bcl-2 expression and decreases Bax expression, which is correlated with the activation of ERK1/2. It is noteworthy that silencing of Bcl-2 expression sensitized BXPC-3 cells to gemcitabine-induced apoptosis and silencing of Bax expression resistant BXPC-3 cells to gemcitabine-induced apoptosis. Blocking ERK1/2 activation by MKK1/2 inhibitor could enhance Gem-induced apoptosis. Taken together, our results suggest that ERK1/2-dependent Bcl-2 upregulation and Bax downregulation are required for drug resistance to gemcitabine in BXPC-3 cells.

ERK1/2 activation results in the phosphorylation of many intracellular proteins that regulate various cellular functions ranging from proliferation and differentiation to apoptosis [21]. Gem can activate ERK1/2 in pancreatic cancer cell types, and activation of ERK1/2 is associated with an increase in cell survival in Gem-treated cells [8,22]. Our study demonstrated that Gem treatment induced ERK1/2 activation, and gemcitabine-induced ERK1/2 activation participates in protection from Gem-mediated apoptosis effect in BXPC-3 cells.

Analysis of Bcl-2 and Bax by immunoblotting subsequent exposure to gemcitabine identified an upregulation of pERK1/2, followed by increased Bcl-2 expression at protein levels and thus, an increase in the Bcl-2/Bax ratio in our study. After pERK1/2 was inhibited by PD98059, the Bcl-2 expression at protein levels was decreased and thus, a decrease in the Bcl-2/Bax ratio. We therefore concluded that Bcl-2/Bax ratio was regulated by pERK1/2.

## Conclusions

We demonstrated in our study that Gem could activate pERK1/2-dependent Bcl-2 upregulation and pERK1/2-dependent Bax dowregulation. Upregulation of Bcl-2 and downregulation of Bax are involved in the mediation of chemotherapy resistance in the BXPC-3 cells. The Bcl-2/Bax ratio is an important cellular survival marker that correlates with responsiveness to drug therapy *in vivo* and *in vitro* [23]. Accordingly, resistance to gemcitabine-induced apoptosis has been described to correlate with an overexpression of antiapoptotic proteins such as the Bcl-2 family [24]. Therefore, our results contribute to a better understanding as to how pERK1/2 inhibitors sensitize cells to gemcitabine-induced apoptosis [8].

Taken together, our results identify a new mechanism showing that the ERK1/2-Bcl-2/Bax signaling pathway is responsible for Gem resistance and suggest that targeting the ERK1/2-Bcl-2/Bax signaling pathway may overcome gemcitabine resistance in human pancreatic cancer.

## References

[1] Squadroni M, Fazio N (2010). Chemotherapy in pancreatic adenocarcinoma. Eur Rev Med Pharmacol Sci..

[2] Burris HA, Moore MJ, Anderson J (1997). Improvements in survival and clinical benefit with gemcitabine as first-line therapy for patients with advanced pancreas cancer: a randomized trial. J Clin Oncol..

[3] Rothenberg ML, Moore MJ, Cripps MC (1996). A phase II trial of gemcitabine in patients with 5-FU-refractory pancreatic cancer. Ann Oncol..

[4] van Riel JMGH, van Groeningen CJ, Pinedo HM (1999). Current chemotherapeutic possibilities in pancreaticobiliary cancer. Ann Oncol..

[5] Kroep JR, Pinedo CJ, van Groeningen CJ (1999). Experimental drugs and drug combinations in pancreatic cancer. Ann Oncol..

[6] Arlt A, Gehrz A, Müerköster S, Vorndamm J, Kruse ML, Fölsch UR (2003). Role of NF-kappaB and Akt/PI3K in the resistance of pancreatic carcinoma cell lines against gemcitabine-induced cell death. Oncogene..

[7] Chadha KS, Khoury T, Yu J, Black JD, Gibbs JF, Kuvshinoff BW, Tan D, Brattain MG, Javle MM (2006). Activated Akt and Erk expression and survival after surgery in pancreatic carcinoma. Ann Surg Oncol.

[8] Zheng C, Jiao X, Jiang Y, Sun S (2013). ERK1/2 activity contributes to gemcitabine resistance in pancreatic cancer cells. J Int Med Res..

[9] Hengartner MO (2010). The biochemistry of apoptosis. Nature..

[10] Lutz RJ (2000). Role of the BH3 (bcl-2 homology 3) domain in the regulation of apoptosis and bcl-2-related proteins. Biochem Soc Trans..

[11] Reed JC (1998). bcl-2 family proteins. Oncogene.

[12] Weinmann P, Bommert K, Mapara MY, Dörken B, Bargou RC (2007). Overexpression of the death-promoting gene bax-alpha sensitizes human BL-41 Burkitt lymphoma cells for surface IgM-mediated apoptosis. Eur J Immunol..

[13] Okamoto K, Ocker M, Neureiter D, Dietze O, Zopf S, Hahn EG, Herold C (2007). bcl-2-specific siRNAs restore gemcitabine sensitivity in human pancreatic cancer cells. J Cell Mol Med.

[14] Schniewind B, Christgen M, Kurdow R, Haye S, Kremer B, Kalthoff H, Ungefroren H (2004). Resistance of pancreatic cancer to gemcitabine treatment is dependent on mitochondria-mediated apoptosis. Int J Cancer..

[15] Pirocanac EC, Nassirpour R, Yang M, Wang J, Nardin SR, Gu J, Fang B, Moossa AR, Hoffman RM, Bouvet M (2002). bax-induction gene therapy of pancreatic cancer. J Surg Res.

[16] Seino S, Sunayama J, Matsuda KI, Sato A, Matsumoto Y, Nomiya T, Nemoto K, Yamashita H, Kayama T, Ando K, Kitanaka C (2002). MEK-ERK-dependent multiple caspase activation by mitochondrial proapoptotic bcl-2 family proteins is essential for heavy ion irradiation-induced glioma cell death. Cell Death Dis..

[17] Zhang W, Zhao L, Liu J, Du J, Wang Z, Ruan C, Dai K (2012). Cisplatin induces platelet apoptosis through the ERK signaling pathway. Thromb Res..

[18] Pan TL, Wang PW, Leu YL, Wu TH, Wu TS (2012). Inhibitory effects of Scutellaria baicalensis extract on hepatic stellate cells through inducing G2/M cell cycle arrest and activating ERK-dependent apoptosis via bax and caspase pathway. J Ethnopharmacol..

[19] Adams JM, Cory S (2007). The bcl-2 apoptotic switch in cancer development and therapy. Oncogene..

[20] Boucher MJ, Morisset J, Vachon PH, Reed JC, Lainé J, Rivard N (2000). MEK/ERK signaling pathway regulates the expression of bcl-2, bcl-X(L), and Mcl-1 and promotes survival of human pancreatic cancer cells. J Cell Biochem..

[21] Peyssonnaux C, Eyche’ne A (2001). The Raf/MEK/ERK pathway: new concepts of activation. Biol Cell..

[22] Tang Y, Liu F, Zheng C, Sun S, Jiang Y (2012). Knockdown of clusterin sensitizes pancreatic cancer cells to gemcitabine chemotherapy by ERK1/2 inactivation. J Exp Clin Cancer Res..

[23] Sartorius UA, Krammer PH (2002). Upregulation of bcl-2 is involved in the mediation of chemotherapy resistance in human small cell lung cancer cell lines. Int J Cancer..

[24] Robertson LE, Plunkett W, McConnell K, Keating MJ, McDonnell TJ (1996). bcl-2 expression in chronic lymphocytic leukemia and its correlation with the induction of apoptosis and clinical outcome. Leukemia.

